# Case Report: Durable complete response of metastatic hepatocellular carcinoma with asymptomatic hyperamylasemia to combined immunotherapy of anti-cytotoxic T lymphocyte-associated antigen 4 plus anti-programmed cell death-1 antibodies

**DOI:** 10.3389/fimmu.2023.1274449

**Published:** 2023-10-06

**Authors:** Han Gao, Rui-zhi Chang, Xiao-ping Chen, Wan-guang Zhang, Bixiang Zhang, Xin Luo, Ze-yang Ding

**Affiliations:** ^1^ Hepatic Surgery Center, Tongji Hospital, Tongji Medical College, Huazhong University of Science and Technology, Wuhan, Hubei, China; ^2^ Clinical Medicine Research Center for Hepatic Surgery of Hubei Province, Tongji Hospital, Tongji Medical College, Huazhong University of Science and Technology, Wuhan, Hubei, China; ^3^ Hubei Key Laboratory of Hepato-Pancreatic-Biliary Diseases, Tongji Hospital, Tongji Medical College, Huazhong University of Science and Technology, Wuhan, Hubei, China

**Keywords:** hepatocellular carcinoma, asymptomatic hyperamylasemia, PD-1 inhibitor, CTLA-4 inhibitor, combined immunotherapy

## Abstract

**Background:**

Combined immunotherapy has shown promising results in the treatment of advanced HCC, whereas the priority population that would respond to the combined immunotherapy is still elusive. In addition, HCC with asymptomatic hyperamylasemia was not reported previously.

**Case presentation:**

An aged patient was diagnosed as HCC with BCLC stage C (bone metastasis). Notably, this patient showed asymptomatic hyperamylasemia. The patient was then enrolled in a trial evaluating combined immunotherapy of anti-PD-1 antibody sintilimab (IBI308) plus anti-CTLA-4 antibody (IBI310) in advanced HCC. After being treated with combined immunotherapy, this patient rapidly achieved complete response (CR) according to mRECIST criteria or immune partial response (iPR) according to iRECIST criteria and maintain the CR state for more than 12 months. Interestingly, serum levels of amylase and lipase in this patient were reduced after treatment.

**Conclusion:**

We reported, for the first time, a case of metastatic HCC with asymptomatic hyperamylasemia, and suggested that HCC patients with asymptomatic hyperamylasemia may benefit from combined immunotherapy of anti-CTLA-4 and PD-1 antibodies.

## Introduction

Hepatocellular carcinoma (HCC) is the sixth most commonly diagnosed cancer and is currently the fourth leading cause of cancer-related death worldwide (1). Currently, immune-checkpoint inhibitors (ICIs) based systemic therapies showed promising effects in treating advanced HCC. Combined immunotherapy of Nivolumab Plus Ipilimumab has been approved as a second-line systemic therapy for advanced HCC, based on the results of the CheckMate 040 trial (2). In addition, the HIMALAYA trial found that the tremelimumab plus durvalumab improved overall survival (16.43 months, 95% CI, 14.16 to 19.58) versus sorafenib (13.77 months, 95% CI, 12.25 to 16.13) (3). In China, a phase 3 trial comparing the effectiveness and safety of anti-cytotoxic T lymphocyte-associated antigen 4 (CTLA-4) antibody (IBI310) combined with sintilimab (IBI308) versus sorafenib in the first-line treatment of advanced HCC is ongoing.

Paraneoplastic syndrome (PNS) was found in 20–40% of HCC patients, and 8–17% of patients having more than one PNS. The most common PNS in HCC are hypercholesterolemia, hypoglycemia, hypercalcemia, and erythrocytosis, whereas rheumatic, neuromuscular, dermatological, hematological, and endocrine syndromes were less commonly found. HCC patients with PNS showed worse prognosis than those without PNS (4), and the best treatment for HCC patients with PNS, especially for those with unresectable, advanced HCC, is still exploring.

In this study, we report a case of metastatic HCC with asymptomatic hyperamylasemia, a PNS that has never been reported in HCC before. This patient showed a significant response to combined immunotherapy of sintilimab plus anti-CTLA-4 antibody.

## Case presentation

In August 2021, an elderly patient was admitted to Tongji Hospital (Wuhan, China) for a mass at the right chest wall with tenderness for 1 month. At admission, this patient received a computed tomography (CT) scan, and results of abdominal CT revealed abnormally enhancing nodules of 92mm and 16 mm in the right posterior lobe and right anterior lobe of the liver respectively. Chest CT revealed a mass in the right anterior chest wall with bone destruction on the right side of the sternum. In addition, the serum level of AFP was 2075 ng/ml at admission. Based on these findings, this patient was diagnosed with HCC according to the clinical diagnostic criteria of AASLD and EASL. The patient’s Eastern Cooperative Oncology Group performance status (PS) score was 0 and liver blood tests at admission showed the patient’s Child-pugh score was 5 (grade A). The patient refused to undergo a biopsy of the tumor for pathological diagnosis. We evaluated the patient’s China Liver Cancer stage (CNLC IIIb: PS 0, Child-Pugh A and extrahepatic metastasis), BCLC stage (BCLC C, PS 0, Child-Pugh A and extrahepatic metastasis) and TNM stage IV (cT3N0M1, multiple tumors, tumor diameter>5cm and distant metastasis), which are critical for treatment choice and prognosis assessment.

**Figure 1:**
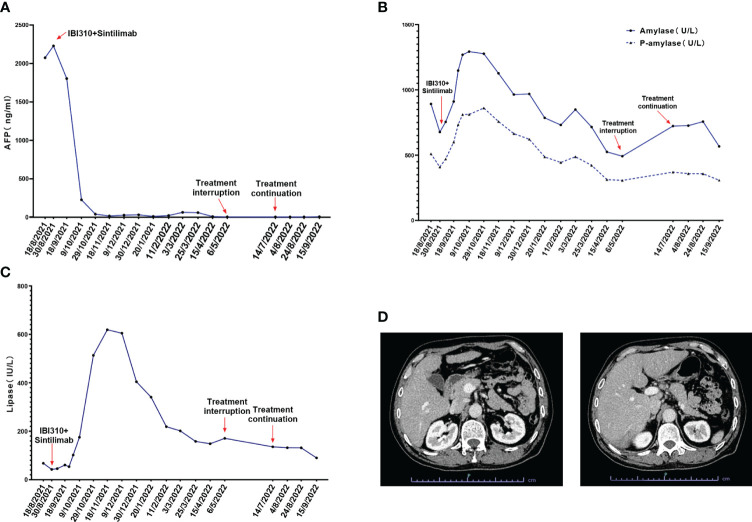
**(A)** The alpha fetoprotein (AFP) levels of the patient during the treatment of sintilimab combined with IBI310; **(B)** The total amylase and P-amylase levels of the patient during the treatment; **(C)** The lipase levels of the patient during the treatment; **(D)** The abdominal CT scan of the patient at admission which revealed no signs of enlargement or inflammation of the pancreas.

**Figure 2:**
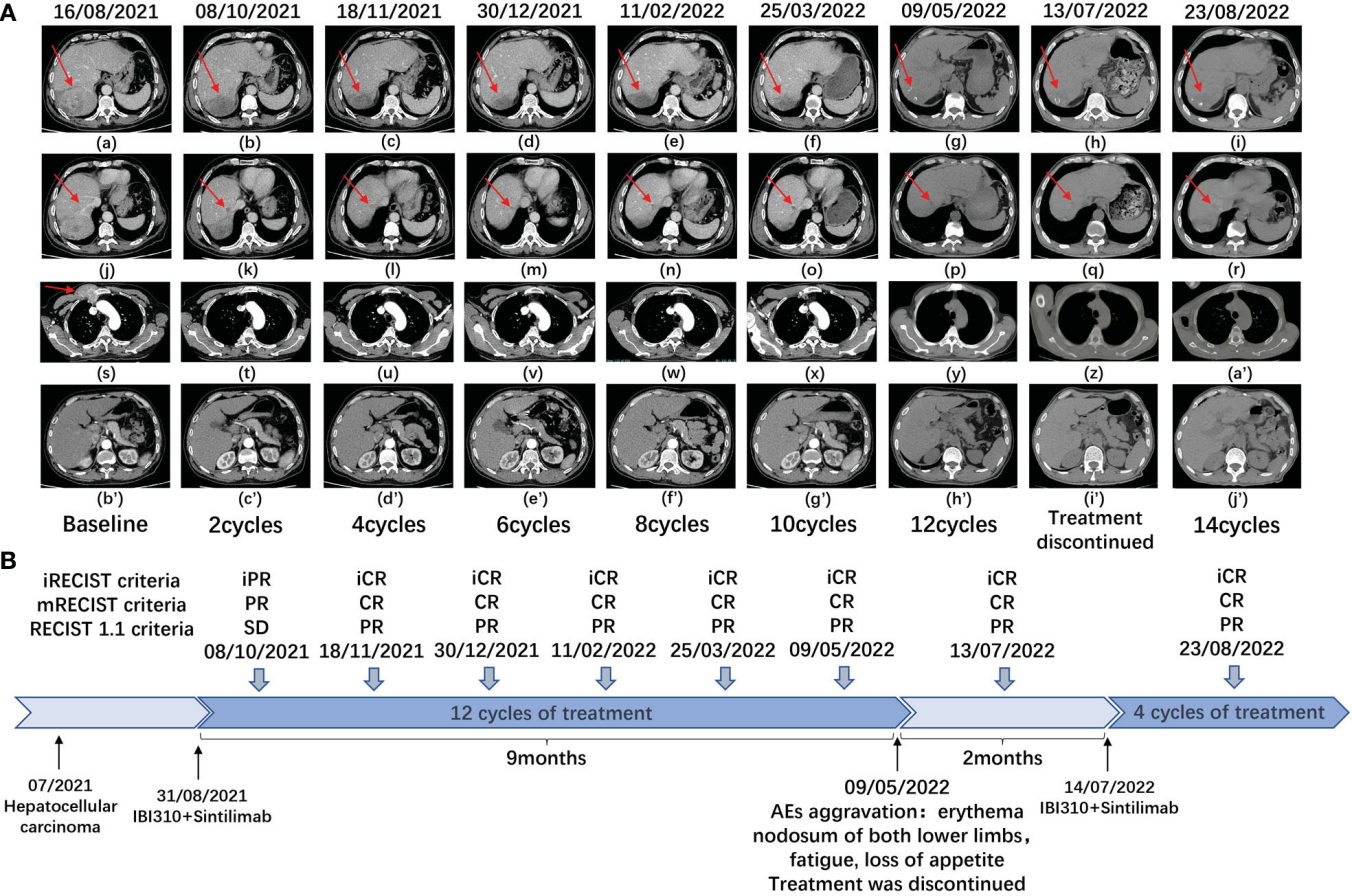
**(A)** Plain and enhanced CT of main lesions of the patient in different periods. a-i: right posterior lobe of the liver; j-r: right anterior lobe of the liver; s-a’ the right anterior chest wall; b’j’: the pancreas with no signs of any inflammation and enlargement. **(B)** This is a flowchart of the treatment of the patient. At different times, the patient received combination immunotherapy (sintilimab plus IBI310). CR, complete response, PR, partial response, SD, stable disease, AEs, adverse events.

After discussion in a multidisciplinary team board meeting based on the evaluation, radical resection was not applicable and systemic therapy with atezolizumab and bevacizumab was recommended to the patient. However, the patient refused this therapy due to its high cost that they could not afford since atezolizumab is not on the list of drugs covered by medical insurance in China. The patient was then recommended to participate in a trial designed to evaluate the efficacy and safety of combined immunotherapy using sintilimab (IBI308) and anti-CTLA-4 antibody (IBI310) versus sorafenib in first-line treatment of advanced HCC (NCT04720716). After signing informed consent form, it was confirmed through screening that this patient met eligibility criteria for trial participation. At screening, it was found that this patient had hyperamylasemia with serum levels of amylase at 892 U/L, pancreatic amylase at 510 U/L, and lipase at 67.7 IU/L. The patient was confirmed to have no symptoms of abdominal pain. The dynamic changes in serum levels of AFP, amylase and lipase are shown in **Figures 1A–C**. The abdominal CT scan revealed no signs of enlargement or inflammation in this patient’s pancreas (**Figure 1D**), and the serum levels of creatinine and eGFR were within normal range. After the screen, this patient was randomized to the group of combined immunotherapy, and began the first dose of combined immunotherapy on August 31st, 2021.The flowchart of the treatment and the changes of the imaging examination is in **Figure 2**. After receiving four cycles of combined immunotherapy, the patient was evaluated as having achieved CR according to mRECIST criteria, iPR according to iRECIST criteria or PR according to RECIST 1.1 criteria. Accordingly, AFP levels decreased rapidly until they eventually returned to normal range. In addition, sternal metastasis was found to have disappeared on physical examination after the third cycle of treatment. Levels of amylase and lipase remained high at the beginning of treatment (1293 U/L and 618.9 IU/L). Interestingly, as the duration of treatment, the levels of amylase and lipase decreased significantly (492 U/L and 89.8 IU/L). Although levels of amylase and pancreatic amylase increased at the first two cycles of immunotherapy, the abdominal CT scan did not reveal any signs of enlargement or inflammation in the pancreas (**Figure 2**).

After beginning combined immunotherapy, the patient subsequently developed a series of adverse events (AEs) (grade 1, CTCAE 5.0), such as fatigue, pruritus, loss of appetite, and hyperglycemia. The patient then developed hypothyroidism and erythema nodosum on both lower limbs (grade 2, CTCAE 5.0) and was treated with levothyroxine and mucopolysaccharide polysulfate cream. At the 12th cycle of treatment (May 9^th^, 2022), the treatment was delayed, since the fatigue and loss of appetite were exacerbated to the grade 3 of CTCAE 5.0 criteria. Treatment continued after these adverse effects were improved to the grade 2 (July 14^th^, 2022). Noticeably, amylase levels rebounded to 723 U/L after the treatment was interrupted. As treatment continued, amylase levels eventually decreased to 567 U/L.

In October 2022, the treatment was delayed again due to a COVID-19 outbreak in Wuhan. After the lift lockdown of the hospital, this patient was informed to continue the treatment, but he refused because he was tired of repeating SARS-CoV-2 tests before every admission, and did not feel any discomfort. The patient received the last dose of combined therapy on September 15^th^, 2022. As of July 2023, telephone follow-ups are still being performed and the patient is surviving without any symptoms.

## Discussion

To the best of our knowledge, this is the first reported case of a patient with advanced HCC and asymptomatic hyperamylasemia. Asymptomatic hyperamylasemia is rarely found in non-pancreatic tumors (5, 6). We performed a literature review of neoplasms associated with hyperamylasemia (**Supplementary Table 1**), and found that it was occasionally present in lung cancer, ovarian cancer, pheochromocytoma, and hematological malignancies including multiple myeloma, MALT lymphoma, AML and ALL (7). Considering that amylase levels are not routinely measured in blood tests during screening and follow-up of HCC, the incidence of HCC with hyperamylasemia might be underestimated. In ALL, hyperamylasemia is usually associated with L-asparaginase toxicity and leukemic infiltration of the pancreas. Besides these situations, amylase-producing tumor cells appear to be the major source of hyperamylasemia in malignancies. In human, α-amylase is encoded by AMY1 (salivary type) and AMY2 (pancreatic type) genes. α-Amylase can be produced by the liver and is encoded by the AMY-2B, an isogene of AMY2 (7, 8). In this case, evidence indicates that elevated levels of amylase may be produced by the tumor. Firstly, both serum levels of amylase and pancreatic amylase were elevated in this case. Secondly, dynamic changes in serum levels of amylase and pancreatic amylase were observed after starting combined immunotherapy. Specifically, levels of amylase and pancreatic amylase increased during the first two cycles of immunotherapy, suggesting that amylase was released from necrotic tumor cells. Levels of amylase decreased during subsequent cycles of immunotherapy. Unfortunately, since the patient refused to undergo a tumor biopsy and histopathological detection of amylase in tumor tissue was unavailable, it was impossible to provide definite evidence for an amylase-producing HCC. Interestingly, levels of amylase behaved like a specific tumor biomarker for predicting efficacy and recurrence in different cancers since its dramatic decrease reflected tumor response to treatment.

In this study, we reported that this case, metastatic HCC with hyperamylasemia showed a quick and durable response to combined immunotherapy. In addition, previous studies have reported that cases of lung cancer, ovarian cancer, and ALL with hyperamylasemia were highly sensitive to chemotherapy (9–11). These results shed light on the association between hyperamylasemia and the sensitivity of cancer patients to systemic therapy including chemotherapy or immunotherapy. Further research is required to understand the underlying mechanisms. Furthermore, this is the first time that combined immunotherapy has been used for the treatment of a malignant tumor with asymptomatic hyperamylasemia. This has shown great predictive potential and efficacy. It remains to be explored whether combined immunotherapy can be generalized to other cancers with asymptomatic hyperamylasemia and whether cancers with asymptomatic hyperamylasemia are more sensitive to immunotherapy.

In conclusion, we reported for the first time a case of metastatic HCC with asymptomatic hyperamylasemia that was highly sensitive to combined immunotherapy using anti-CTLA-4 and anti-PD-1 antibodies. Our report suggests that despite being rarely found in HCC, hyperamylasemia might serve as a marker for reflecting the efficacy of immunotherapy. This association is worth validating in a large HCC cohort in the future.

## Data availability statement

The original contributions presented in the study are included in the article/**Supplementary Material**. Further inquiries can be directed to the corresponding authors.

## Ethics statement

The studies involving humans were approved by ethics committee of Huazhong University of Science and Technology. The studies were conducted in accordance with the local legislation and institutional requirements. The participants provided their written informed consent to participate in this study. Written informed consent was obtained from the individual(s) for the publication of any potentially identifiable images or data included in this article. Written informed consent was obtained from the participant/patient(s) for the publication of this case report.

## Author contributions

HG: Writing – original draft, Writing – review & editing, Methodology. R-ZC: Investigation, Writing – original draft, Writing – review & editing. X-PC: Supervision, Writing – review & editing. W-GZ: Supervision, Writing – review & editing. BZ: Supervision, Writing – review & editing. XL: Supervision, Writing – review & editing. Z-YD: Funding acquisition, Supervision, Writing – review & editing.
