# Supplementary material for: Case Report: Durable complete response of metastatic hepatocellular carcinoma with asymptomatic hyperamylasemia to combined immunotherapy of anti-cytotoxic T lymphocyte-associated antigen 4 plus anti-programmed cell death-1 antibodies
Source: Front Immunol. 2023 Oct 6;14:1274449. doi: 10.3389/fimmu.2023.1274449 (PMC10587582; doi:10.3389/fimmu.2023.1274449)
Supplement: Supplementary file 1 [file Table_1.docx]

Supplementary Table 1. A literature review of case reports of neoplasms associated with hyperamylasemia.

| References | Type of malignancy | Pathology | Treatment | Outcome | Results of serum amylase levels after treatment |
| --- | --- | --- | --- | --- | --- |
| [1] | Lung cancer | LUAD | Radiotherapy | Unknown | Unknown |
|  | Lung cancer | SCLC | Unknown | Died of disease relapse | Unknown |
| [2] | Lung cancer | LUAD | Symptomatic treatment | Died of disease relapse | Unknown |
| [3] | Lung cancer | LUAD | Undone | died of respiratory insufficiency | Unknown |
| [4] | Lung cancer | LUAD | Radiotherapy | Died of apnea and absent pulse | Unknown |
| [5] | Lung cancer | LUAD | Unknown | Died of respiratory failure | Unknown |
| [6] | Lung cancer | LUAD | Carboplatin, paclitaxel, gefitinib | Died of disease relapse | Paralleled the LUAD response to treatment and disease relapse |
| [7] | Lung cancer | LUAD | Chemotherapy | Died of disease relapse | Unknown |
| [8] | Lung cancer | LUAD | Gefitinib, radiotherapy | Died of respiratory failure | Decreased to normal |
| [9] | Lung cancer | SCLC | Cisplatin and etoposide | BOR is CR, TTR is 3 weeks | Decreased to normal |
| [10] | Lung cancer | SCLC | Carboplatin, etoposide, radiotherapy | Died of disease relapse | Paralleled the SCLC response to treatment and disease relapse |
| [11] | Lung cancer | SCLC | Undone | Died of cardiopulmonary failure | Unknown |
| [12] | Lung cancer | SCLC | Carboplatin, etoposide | BOR is CR, TTR is 3 weeks | Decreased to normal |
| [13] | Lung cancer | SCLC | Carboplatin, irinotecan | Died of disease relapse | Paralleled the SCLC response to treatment and disease relapse |
| [14] | Ovary cancer | OV | Surgery | No recurrence until the last follow-up | Decreased to normal |
| [15] | Ovary cancer | OV | Surgery | No recurrence until the last follow-up | Decreased to normal |
| [16] | Ovary cancer | OV | Unknown | Died of disease relapse | No significant improvement |
| [17] | Ovary cancer | OV | Paclitaxel, carboplatin | Favorable prognosis | Decreased significantly |
| [18] | Ovary cancer | OV | Surgery, paclitaxel, carboplatin | Unknown | Paralleled the OV response to treatment and disease progression |
| [19] | Ovary cancer | OV | Surgery | No recurrence until the last follow-up | Unknown |
| [20] | Ovary cancer | OV | Surgery, paclitaxel, carboplatin, gemcitabine, capecitabine | Unknown | Decreased to normal |
| [21] | Neuroendocrine neoplasm | PCPG | Surgery | No recurrence until the last follow-up | Decreased to normal |
| [22] | Neuroendocrine neoplasm | PCPG | Surgery | No recurrence until the last follow-up | Decreased to normal |
| [23] | Hematologic malignancy | MM | Melphalan, prednisone, bortezomib | Died of MM | Paralleled the MM response to treatment and disease progression |
| [24] | Hematologic malignancy | MM | Vincristine, doxorubicine, dexamethasone | Died of MM | Paralleled the MM response to treatment and disease progression |
| [25] | Hematologic malignancy | MM | Idorubicin, dexamethasone, bone marrow allograft | Died of disease relapse | Decreased to normal |
| [26] | Hematologic malignancy | MM | adriamycin, dexamethasone, prednisone, bortezomib | Died of MM | No significant improvement |
| [27] | Hematologic malignancy | MM | vincristin, Adriamycin, dexamethasone, bone marrow transplant | Died of disease relapse | Paralleled the MM response to treatment and disease progression |
| [28] | Hematologic malignancy | MM | Dexamethasone, thalidomide, bortezomib, dexamethasone | BOR is CR, TTR is 18 weeks | Decreased to normal |
| [29] | Hematologic malignancy | MM | Melphalan, prednisone | Died of renal failure | No significant improvement |
| [30] | Hematologic malignancy | MM | Vindesine, idarubicin, dexamethasone, melphalan, prednisone | Died of disease relapse | No significant improvement |
| [31] | Hematologic malignancy | MALT | Surgery | Unknown | Unknown |
| [32] | Hematologic malignancy | ALL | Vincristine, daunorubicin, L-asparaginase, methotrexate, imatinib | BOR is PR, TTR is 3 weeks | Decreased to normal |
| [33] | Hematologic malignancy | AML | Chemotherapy | BOR is CR, TTR is unknown | Decreased to normal |

TTR (time to recurrence) for patients received curative surgery, TTR (time to response) * for patients received systemic therapy, BOR (best of response) for patients received systemic therapy, CR, complete response, PR, partial response, LUAD, lung adenocarcinoma, SCLC, small cell lung cancer, OV, ovarian serous cystadenocarcinoma, PCPG, pheochromocytoma and paraganglioma, MM, multiple myeloma, MALT, mucosal-associated lymphoid tissue, ALL, acute lymphoblastic leukemia, AML, acute myeloid leukemia

References:

1. Ammann RW, Berk JE, Fridhandler L, Ueda M, Wegmann W: Hyperamylasemia with carcinoma of the lung. Ann Intern Med 1973, 78(4):521-526.

2. Weiss MJ, Edmondson HA, Wertman M: Elevated serum amylase associated with bronchogenic carcinoma; report of case. Am J Clin Pathol 1951, 21(11):1057-1061.

3. Gomi K, Kameya T, Tsumuraya M, Shimosato Y, Zeze F, Abe K, Yoneyama T: Ultrastructural, histochemical, and biochemical studies of two cases with amylase, ACTH, and beta-MSH producing tumor. Cancer 1976, 38(4):1645-1654.

4. Martin PC, Sarma DP: Amylase-producing lung cancer. J Surg Oncol 1982, 21(1):30-32.

5. Yokoyama M, Natsuizaka T, Ishii Y, Ohshima S, Kasagi A, Tateno S: Amylase-producing lung cancer: ultrastructural and biochemical studies. Cancer 1977, 40(2):766-772.

6. Minami S, Jokoji R, Yamamoto S, Ogata Y, Koba T, Futami S, Nishijima Y, Yaga M, Masuhiro K, Tsujimoto M et al: Amylase-Producing Lung Cancer with a Positive Epidermal Growth Factor Receptor Mutation Treated With Gefitinib: A Case Report. World J Oncol 2014, 5(1):41-46.

7. Katayama S, Ikeuchi M, Kanazawa Y, Akanuma Y, Kosaka K, Takeuchi T, Nakayama T: Amylase-producing lung cancer: case report and review of the literature. Cancer 1981, 48(11):2499-2502.

8. Ko HW, Tsai YH, Yu CT, Huang CY, Chen CH: Good response to gefitinib for lung adenocarcinoma with hyperamylasemia: a case report. Chang Gung Med J 2008, 31(6):606-611.

9. Wang H, Wu Q: A case of amylase-producing small cell lung cancer. Clin Biochem 2016, 49(7-8):613-616.

10. Benedetti G, Rastelli F, Damiani S, Calandri C, Crinò L: Challenging problems in malignancy: case 1. Presentation of small-cell lung cancer with marked hyperamylasemia. J Clin Oncol 2004, 22(18):3826-3828.

11. Cekerevac I, Petrović M, Novković L, Bubanja D, Bubanja I, Djokić B, Stanković V, Jurisić V: ECTOPIC ACTH SECRETION WITH CONCOMITANT HYPERAMYLASEMIA IN A PATIENT WITH SMALL CELL LUNG CARCINOMA: CASE REPORT. Acta Clin Croat 2015, 54(4):536-540.

12. Akinosoglou K, Siagris D, Geropoulou E, Kosmopoulou O, Velissaris D, Kyriazopoulou V, Gogos C: Hyperamylasaemia and dual paraneoplastic syndromes in small cell lung cancer. Ann Clin Biochem 2014, 51(Pt 1):101-105.

13. Yanagitani N, Kaira K, Sunaga N, Naito Y, Koike Y, Ishihara S, Ishizuka T, Saito R, Mori M: Serum amylase is a sensitive tumor marker for amylase-producing small cell lung cancer? Int J Clin Oncol 2007, 12(3):231-233.

14. Srivastava R, Fraser C, Gentleman D, Jamieson LA, Murphy MJ: Hyperamylasaemia: not the usual suspects. Bmj 2005, 331(7521):890-891.

15. Yagi C, Miyata J, Hanai J, Ogawa M, Ueda G: Hyperamylasemia associated with endometrioid carcinoma of the ovary: case report and immunohistochemical study. Gynecol Oncol 1986, 25(2):250-255.

16. Hayakawa T, Kameya A, Mizuno R, Noda A, Kondo T, Hirabayashi N: Hyperamylasemia with papillary serous cystadenocarcinoma of the ovary. Cancer 1984, 54(8):1662-1665.

17. Tohya T, Shimajiri S, Onoda C, Yoshimura T: Complete remission of ovarian endometrioid adenocarcinoma associated with hyperamylasemia and liver metastasis treated by paclitaxel and carboplatin chemotherapy: a case report. Int J Gynecol Cancer 2004, 14(2):378-380.

18. Logie JJ, Cox M, Sharkey J, Williams A: A multidisciplinary approach to an unusual cause of hyperamylasaemia. BMJ Case Rep 2015, 2015.

19. Burden S, Poon AS, Masood K, Didi M: Hyperamylasaemia: pathognomonic to pancreatitis? BMJ Case Rep 2013, 2013.

20. Guo S, Lv H, Yan L, Rong F: Hyperamylasemia may indicate the presence of ovarian carcinoma: A case report. Medicine (Baltimore) 2018, 97(49):e13520.

21. Ho ET, Gardner DS: Paraganglioma with acute hyperamylasaemia masquerading as acute pancreatitis. Singapore Med J 2011, 52(12):e251-254.

22. Wu HW, Liou WP, Chou CC, Chen YH, Loh CH, Wang HP: Pheochromocytoma presented as intestinal pseudo-obstruction and hyperamylasemia. Am J Emerg Med 2008, 26(8):971.e971-974.

23. Calvo-Villas JM, Alvarez I, Carretera E, Espinosa J, Sicilia F: Paraneoplastic hyperamylasaemia in association with multiple myeloma. Acta Haematol 2007, 117(4):242-245.

24. Kaneko H, Ohkawara Y, Taniguchi K, Matsumoto Y, Nomura K, Horiike S, Yokota S, Taniwaki M: Simultaneous complication of multiple myeloma with Sjögren syndrome. Asian Pac J Allergy Immunol 2006, 24(4):245-248.

25. Ross CM, Devgun MS, Gunn IR: Hyperamylasaemia and multiple myeloma. Ann Clin Biochem 2002, 39(Pt 6):616-620.

26. García Martín F, Huerta Arroyo A, Alcázar De La Ossa JM, Praga Terente M: [Hyperamylasaemia associated with multiple myeloma on haemodialysis]. Nefrologia 2009, 29(1):93-94.

27. Roux CH, Breuil V, Brocq O, Albert C, Chami H, Euller-Ziegler L: Hyperamylasemia and back pain: an exceptional association revealing multiple myeloma. Joint Bone Spine 2007, 74(1):112-113.

28. Nafil H, Tazi I, Mahmal L: [A rare cause of hyperamylasemia]. Ann Biol Clin (Paris) 2012, 70(2):237-238.

29. Machida T, Shizuka R, Yabe S, Kobayashi Y, Yomoda S, Sawamura M, Murakami M: Identification of amylase-binding monoclonal immunoglobulins in multiple myeloma associated with macroamylasemia. Leuk Lymphoma 2012, 53(11):2293-2295.

30. Sagristani M, Guariglia R, Pocali B, De Rienzo M, Guastafierro S, Romano G, Tirelli A: Macroamylasemia in a patient with multiple myeloma. Leuk Lymphoma 2002, 43(8):1705-1707.

31. Ogasawara N, Imamura T, Sato Y, Tamura T, Urasaki Y, Kono Y, Koyama R, Inoshita N, Nishida A: Simultaneous macroamylasemia and macrolipasemia in a patient with mucosa-associated lymphoid tissue lymphoma. Clin J Gastroenterol 2020, 13(4):626-631.

32. Guruprasad CS, Reghu KS, Nair M, Kumary PK: Asymptomatic Hyperamylasemia / Hyperlipasemia due to Pancreatic Infiltration in Acute Lymphoblastic Leukemia. Indian J Pediatr 2016, 83(1):81-82.

33. Nakayama S, Yokote T, Kobayashi K, Hirata Y, Akioka T, Miyoshi T, Takubo T, Tsuji M, Hanafusa T: Macroamylasemia in a patient with acute myeloid leukemia. Leuk Res 2009, 33(8):e121-123.
